# *Talin 2* is a large and complex gene encoding multiple transcripts and protein isoforms

**DOI:** 10.1111/j.1742-4658.2009.06893.x

**Published:** 2009-03

**Authors:** Emmanuel Debrand, Yasmine El Jai, Lorraine Spence, Neil Bate, Uta Praekelt, Catrin A Pritchard, Susan J Monkley, David R Critchley

**Affiliations:** Department of Biochemistry, University of LeicesterUK

**Keywords:** cytoskeleton, integrins, spermatogenesis, talin 2, talin 2 isoforms

## Abstract

Talins are large adaptor proteins that link the integrin family of adhesion molecules to F-actin. In vertebrates, there are two talin genes. *Talin 1* is essential for integrin-mediated cell adhesion; the role of *talin 2* is unclear. Here we report a detailed analysis of mammalian *talin 2*. This reveals the existence of a previously unrecognized promoter associated with a CpG island, and separated from the first coding exon by numerous alternatively spliced noncoding exons spanning > 200 kb. Analysis of a mouse gene trap line shows that this promoter accounts for most of the talin 2 expression in adult tissues. We also demonstrate that testis and kidney express truncated talin 2 isoforms that lack the N-terminal half of the protein, and provide evidence for the developmentally regulated expression of the short testis-specific talin 2 isoform in elongating spermatids. Finally, we identify four tissue-specific alternative splicing events within the coding region of *talin 2*.

Talin is a large (∼ 270 kDa, ∼ 2540 amino acids) dimeric cytoskeletal protein that couples the integrin family of αβ heterodimeric cell adhesion molecules to the actin cytoskeleton [1]. It also plays a key role in regulating the affinity of integrins for their ligands [2]. Talin contains an N-terminal FERM domain (residues 1–400) that contains binding sites for β-integrin cytoplasmic domains [3], F-actin [4], the adaptor protein Wech [5] and also PIP-kinase type 1γ [6,7], which is part of the signalling pathway that regulates assembly of integrin-mediated cellular junctions with the extracellular matrix (ECM). The FERM domain is linked to a large flexible rod that contains a second integrin-binding site [8], at least two actin-binding sites [9], and multiple binding sites for the cytoskeletal protein vinculin [10], which is involved in stabilizing cell–ECM junctions [11].

There are two talin genes in vertebrates, which have conserved intron–exon boundaries and encode very similar proteins (74% amino acid sequence identity) [12,13]. However, *talin 1* has relatively small introns resulting in a gene of ∼ 30 kb, whereas *talin 2* is much bigger (coding sequence ∼ 190 kb), owing to the presence of larger introns. *Talin 2* is predicted to be the ancestral gene, and it appears to have undergone duplication in chordates prior to the emergence of vertebrates to produce *talin 1* [13]. Talin gene duplication also occurred in the Amboebozoa, and *Dictyostelium discoideum* has two talin genes, *TalA* and *TalB*, which encode proteins with distinct functions: TalA is required for cell–substrate adhesion, phagocytosis and cytokinesis, whereas TalB is required for the force transmission required to support morphogenetic movements during differentiation [14].

Studies in mammalian systems have largely focused on talin 1, which is ubiquitously expressed, and is implicated in a wide variety of integrin-mediated events. At the cellular level, disruption of both *talin 1* alleles (*Tln1*) in mouse ES cells confirms previous findings that talin 1 is required for the assembly of integrin-containing cell–ECM junctions (focal adhesions) [15]. However, in lymphoid cells, integrins also mediate cell–cell interactions, and talin 1 is required for T-cell receptor-mediated clustering and polarization of integrin αLβ2 (lymphocyte function-associated antigen-1) and the regulation of its affinity for intercellular adhesion molecule-1, and for the assembly of the immunological synapse [16]. Talin 1 also regulates the affinity of lymphocyte integrin α4β1 (very late antigen-4) for vascular cell adhesion molecule-1 on endothelial cells, which is important for lymphocyte trafficking [17], and in macrophages, talin 1 is required for phagocytosis mediated by αMβ2 integrin [18]. In mice, disruption of both *Tln1* alleles results in embryonic lethality at 8.5–9.5 days *post coitum*, owing to gastrulation defects [19], and more recent studies, using a conditional *Tln1* allele, have demonstrated that talin 1 is required for the activation of platelet integrins *in vivo* [20,21], for the integrity of the myotendinous junction in skeletal muscle [22], and for the association of the plasma membrane with the cytoskeleton in megakaryocytes [23].

The role of talin 2 is much less clear. Northern blotting initially suggested that in the mouse, *Tln2* expression was more restricted than *Tln1* expression, and *Tln2* mRNAs were most abundant in heart, brain and skeletal muscle [12]. However, more recent western blotting data and expression studies with a mouse gene trap line suggest that *Tln2* may be more widely expressed [24,25]. The interpretation of published immunocytochemical studies on the expression and cellular localization of talin in tissues is complicated by the fact that many of the commonly used talin antibodies cross-react with both proteins, although studies with isoform-specific antibodies have recently been published. Talin 2, but not talin 1, was localized to the costameres and intercalated discs in cardiomyocytes [25], whereas talin 1 and talin 2 were both localized in the myotendinous junction, which may explain why mice with a muscle-specific inactivation of *Tln1* have an only mildly dystrophic phenotype [26]. Talin 2 is reportedly the most abundant isoform in brain [25], and is found in the synapse, where a talin 2–PIP-kinase type 1γ complex is thought to play a role in clathrin-mediated endocytosis [27]. Surprisingly, mice homozygous for a *Tln2*β-Geo gene trap inserted in intron 28 are viable and fertile [24], suggesting either that talin 2 is not essential or that the talin 2–β-galactosidase fusion protein contains the key functional elements of the talin 2 protein. Northern blot data also suggest that the gene trap insertion only ablates *Tln2* mRNA in a subset of tissues, such as heart and testis, as some residual expression is detected in other tissues, e.g. brain and kidney [24]. Thus, splicing out of the gene trap may well lead to expression of low levels of wild-type talin 2. A completely null allele will be required to address these issues.

Most mouse tissues express several large *Tln2* transcripts, ranging in size from 7 to 10 kb, and smaller *Tln2* transcripts have been detected in testis (4.8 kb) and kidney (3.9 kb), although these are too short to encode the full-length protein [12,24]. In order to fully characterize the structure of *talin 2*, to understand the origins and functions of its multiple transcripts, and to inform the design of future knockout and knockdown experiments, we report here a detailed analysis of *Tln2* based on *in silico*, RT-PCR and functional genomic approaches.

## Results

### Mouse *Tln2* spans 414 kb and contains multiple 5′ noncoding exons

**Fig. 1:**
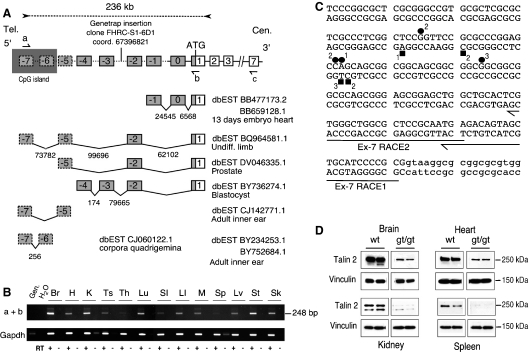
The 5′-end of mouse *Tln2* is associated with a CpG island and contains a large number of 5′-UTR exons scattered over 200 kb. (A) Schematic diagram of the 5′-region of mouse *Tln2* and corresponding ESTs. *Tln2* is transcribed from the minus strand; consequently, the mouse chromosome 9 telomere is on the left (Tel.) and the centromere is on the right (Cen.). Boxes represent previously known exons; dashed boxes denote new putative exons and are linked to the rest of *Tln2* by a dashed line. The first codon (ATG) is in exon 1, which contains both noncoding and coding sequences. Noncoding exons (grey) are numbered from 0 to −7. The CpG island associated with exon −7 is represented by a grey rectangle. The identity and position of the gene trap insertion downstream of exon −3 are indicated. Below the genomic structure, ESTs are shown that contain different combinations of *Tln2* exons. The distance between the exons is indicated in bp, and the oblique lines represent splicing events. (B) RT-PCR between exon −7 and exon 1 (primers *a* + *b*) revealed widespread expression of *Tln2* from the CpG island region in adult mouse tissues. Gen., genomic DNA; H_2_O, negative water control; RT+, reversed-transcribed RNA; RT−, non-reversed transcribed control. *Gapdh* primers were used to assess the amount of cDNA used in PCR reactions. Br., brain; H., heart; K, kidney; Ts, testis; Th, thymus; Lu, lung; SI, small intestine; LI, large intestine; M, skeletal muscle; Sp, spleen; Lv, liver; St, stomach; Sk, skin. (C) 5′-RACE anchored in exon −7 confirmed the 5′-end of *Tln2*. The sequence of exon −7 and flanking 5′ and 3′ (lower-case) sequences are shown. The position of the RACE primers in exon −7 is indicated by half-arrows. The RACE products obtained were cloned and sequenced. The position of the transcriptional start sites is indicated by squares and circles in brain and kidney respectively. The number of clones obtained at each position is also shown. (D) The gene trap insertion downstream of exon −3 dramatically reduces levels of talin 2 in mouse adult tissues. Proteins from wild-type (wt) and homozygous gene trap (gt/gt) mice were analysed by western blotting. Equal amount of total proteins were loaded, and talin 2 was detected with a monoclonal antibody against talin 2 (epitope within residues 482–991). Vinculin was used as an additional loading control and as a normalization reporter to quantify the reduction in Tln2 expression by luminometry (% of residual talin 2). The reduction in talin 2 levels was ∼ 80% in brain, 63% in heart, 93% in kidney, and 97% in spleen.

Initial studies on human and mouse *talin 1* and *talin 2* showed that, although they share the same genomic structure, *talin 2* is a much larger gene, owing to the larger size of the *talin 2* introns [12,13]. Analysis of mouse expressed sequence tags (ESTs) and cDNAs covering the 5′-end of mouse *Tln2* now reveals an additional eight 5′-exons spanning 236 kb (Fig. 1A and Table S2). These exons do not encode any ORF in-frame with the rest of the *Tln2* coding sequence, and to reflect the absence of coding potential, we numbered them exon −7 (most 5′) to exon 0 with respect to the first known coding exon (exon 1). The two most 5′ exons are embedded in a 1.45 kb CpG island (Fig. 1A). To confirm the presence of transcripts containing both the first coding exon (exon 1) and the most 5′ exon (exon −7), we used RT-PCR on mRNAs isolated from 13 tissues. Sequencing of the 248 bp amplicon detected in all tissues (Fig. 1B) revealed that it contains exon −7, exon −5, exon −2 and exon 1; this is identical to the combination found in EST BQ964581.1 (Fig. 1A). Other alternatively spliced transcripts were expressed at lower levels in some tissues, e.g. brain (Figs 1B and S1). These results: (a) show that previously uncharacterized alternatively spliced 5′-exons are present in *Tln2* transcripts; (b) suggest that they originate from a new ubiquitous promoter lying within a CpG island; and (c) demonstrate that *Tln2* is much larger (∼ 414 kb) than previously thought.

In order to establish that *Tln2* does not extend further in the 5′ direction, we generated RACE cDNA libraries using total RNA from mouse brain and heart. Two rounds of amplification with nested primers in exon −7 revealed the presence of a diffuse 150 bp fragment compatible with a transcription start site in or near the CpG island (data not shown). Cloning and sequencing of the PCR products confirmed that the 5′-end of the *Tln2* transcripts lies within the CpG island, although the transcription start site varied slightly within the same tissue and also between tissues (Fig. 1C). This may reflect the absence of a TATA-box, and suggests that the *Tln2* promoter associated with the CpG island is a housekeeping promoter that relies on the positioning of various combinations of transcription factors (TFs) for transcription initiation [28,29]. Analysis of the 5′-end of human *TLN2* confirms that, as in mouse, transcription is driven from a CpG island situated ∼ 200 kb away from the first coding exon, and that the 5′-UTR exons, some of which are conserved between mouse and human, show complex patterns of alternative splicing in different cell types (Table 1 and Fig. S2).

**Table 1:** Conservation of 5′-UTR *talin 2* exons in humans. −, minus strand; +, plus strand; ND, not detected; NA, not applicable.

Mouse	Location on chromosome 9	Distance to first coding exon (bp)	Conservation on human chromosome 15	Distance to first coding exon in humans (bp)
CpG island	67 480 506–67 481 954	236 635	60 469 795–60 471 688	255 081
Exon −7	67 481 698–67 481 838 (−)	236 519	60 470 017–60 470 174 (+)	256 595
Exon −6	67 480 721–67 481 441 (−)	236 122	60 470 426–60 471 217 (+)	255 552
Exon −5	67 407 421–67 407 510 (−)	162 191	60 555 817–60 555 905 (+)	170 864
Exon −4	67 387 385–67 387 419 (−)	142 100	ND	NA
Exon −3	67 387 139–67 387 210 (−)	141 891	ND	NA
Exon −2	67 307 723–67 307 796 (−)	62 477	60 669 179–60 669 251 (+)	57 518
Exon −1	67 276 569–67 276 610 (−)	31 291	ND	NA
Exon 0	67 252 059–67 252 157 (−)	6838	60 716 850–60 716 923 (+)	9846
Exon 1	67 245 319–67 245 490 (−)	NA	60 726 769–60 726 937 (+)	NA
ATG	67 245 487 (−)	NA	60 726 802 (+)	NA

### *In silico* identification of a conserved minimal promoter associated with the CpG island

As the CpG island is conserved in other mammals (data not shown), we sought to establish whether a conserved promoter is present in the vicinity. We used two different *in silico* methods (frameworker and dialign tf; Genomatix) to analyse a 2.2 kb segment upstream of exon −7 in six different mammalian species (Fig. S3 and Experimental procedures). Both approaches revealed the presence of the same highly conserved cluster of TF-binding sites just upstream of the transcriptional start sites identified by 5′-RACE in the mouse CpG island. The arrangement and position of these sites is strictly conserved in six different species, and they are predicted to recruit ubiquitously expressed TFs, such as nuclear respiratory factor 1, elongation factor 2 (E2F), cAMP-responsive element binding protein and E-twenty-six (ETS), which are associated with a large number of ubiquitously expressed housekeeping genes [30]. These results strongly suggest that a core promoter is embedded within the CpG island at the 5′-end of the mammalian gene, and that its use underlies the ubiquitous expression of *Tln2* transcripts (Fig. 1B). Furthermore, examination of chromatin modification data supplied with the latest human genome build (ensembl v50; Fig. S4) strongly supports the idea of a promoter associated with the CpG island in humans. Indeed, it indicates that a 298 bp region overlapping our minimal promoter bears chromatin features characteristics of a promoter, i.e. presence of histone H2AZ, H3K9 monomethylation, H3K4 dimethylation and trimethylation, and DNAse I hypersensitivity (Fig. S4). It also suggests that, at least in humans, no other sequences upstream of the first coding exon exhibit the same features, and therefore that a single promoter may control the expression of *TLN2*.

### Transcription from the CpG island accounts for the majority of *Tln2* expression in adult tissues

To get a better insight into the functional significance of the CpG island and its associated promoter, we analysed mice carrying a gene trap insertion in the intron downstream of exon −3 (Fig. 1A and Experimental procedures), which should interrupt transcription from the CpG region. Mice homozygous for the gene trap (gt/gt) were viable, and we therefore analysed talin 2 levels in various tissues (brain, heart, kidney and spleen) by western blotting (Fig. 1D). Strikingly, the gene trap insertion led to a dramatic reduction in talin 2 levels in all four tissues (Fig. 1D). This indicates that the region upstream of exon −3 contains one or several major site(s) of transcription initiation. It also strongly suggests that the promoter identified in this study is a major *Tln2* promoter. However, the gene trap failed to completely ablate talin 2 expression. This could be due to the intrinsic properties of gene traps, as skipping of gene trap cassettes and alternative splicing around the transgene have previously been described [31–33]. Alternatively, it is possible that other *Tln2* promoters exist downstream of the gene trap insertion. Nevertheless, the extent of the reduction in talin 2 level observed strongly suggests that transcription upstream of the gene trap, most likely from the promoter in the CpG island, is responsible for the majority of talin 2 production in adult tissues.

### A truncated talin 2 is expressed in elongating spermatids of the mature testis

**Fig. 2:**
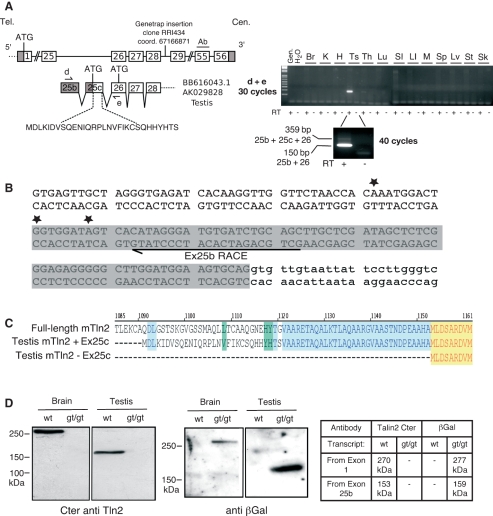
A shorter *Tln2* transcript and protein product are specifically expressed in testis. (A) Schematic diagram of the mouse genomic region between coding exons 25 and 56, showing the gene trap insertion downstream of exon 28. The two testis ESTs containing additional exons 25b and 25c are shown under the genomic region. Grey boxes represent noncoding exons; white boxes are coding sequences. The additional amino acid sequence encoded by exon 25c is indicated. The position of the epitope recognized by the C-terminal polyclonal and monoclonal antibodies (Ab) is shown (encoded by exon 55). Half-arrows represent primers used in RT-PCR. RT-PCR amplification after 30 cycles and 40 cycles (testis) using primers *d* + *e* are depicted on the right-hand side. Gen., genomic DNA; H_2_O, negative water control; RT+, reversed-trascribed RNA; RT−, non-reversed-transcribed control. Br, brain; H, heart; K, kidney; Ts, testis; Th, thymus; Lu, lung; SI, small intestine; LI, large intestine; M, skeletal muscle; Sp, spleen; Lv, liver; St, stomach; Sk, skin. (B) 5′-RACE confirms the 5′-end of short testis-specific *Tln2* transcripts. The sequence of exon 25b is shown – the position of ESTs is highlighted in grey; the position of the RACE primer is indicated by a half-arrow; and the position of the 5′-ends of three sequenced RACE amplicons is marked by a star. (C) Alignment of the predicted N-termini of the truncated testis-specific talin 2 isoforms with the full-length talin 2. +Ex25C, translation of transcripts containing exon 25c using the in-frame ATG present in exon 25c; −Ex25c, translation of short transcripts in which exon 25c is skipped (exons 25b + 26 to 56), using the next ATG in exon 26. The colour wrapping indicates identical amino acids in two (blue) or three (yellow) sequences; green represents positions occupied by different but structurally similar amino acids. (D) Western blotting of protein extracts from wild-type (wt) and homozygous gene trap (gt/gt) mice. Left panel: probed with a C-terminal polyclonal anti-talin 2 antibody [see (A) and Experimental procedures]. Right panel: probed with a polyclonal antibody against β-galactosidase. Br, brain; Ts, testis. The table indicates the predicted molecular masses of proteins detected by the two antibodies.

Northern blots of testis mRNA reveal a 4.8 kb *Tln2* transcript that is too small to encode the full-length protein [12,24]. A testis EST containing two new 5′-exons (25b and 25c; Fig. 2 and Table S2) and predicted to encode a talin 2 lacking the talin head and half of the rod domain has also been described [24]. To analyse the expression pattern of exons 25b and 25c, we performed RT-PCR on a panel of tissues with primers located in exons 25b and 26 (Fig. 2A). This revealed high-level expression of exon 25b specifically in testis and very low levels in thymus. Surprisingly, the main RT-PCR amplicon in testis is too short to contain exons 25b, 25c and 26, although a minor larger product was also observed (Fig. 2A, 40 cycles). Both products were sequenced, and this revealed that skipping of exon 25c occurs in a proportion of transcripts.

Exon 25b is predicted to be noncoding, whereas exon 25c harbours 84 bp of noncoding sequence and an ATG at position 85–87 in-frame with the full-length *Tln2* ORF [24]. Use of this ATG would add a unique 30 amino acid sequence to the N-terminus of this truncated talin 2 isoform, which otherwise would be identical to residues 1121–2542 of the C-terminal half of full-length talin 2. However, the above RT-PCR data suggest that a second protein product may result from the skipping of exon 25c. In this case, the transcript would contain a unique 5′-UTR, and the next in-frame ATG would be located in exon 26. Consequently, the protein would not contain the N-terminal 30 amino acid extension (Fig. 2C), and would be equivalent to residues 1153–2541 of full-length talin 2. To investigate whether exon 25b encodes the 5′-end of the short testis transcript, we first carried out quantitative RT-PCR. This showed that *Tln2* transcripts possessing sequences downstream of exons 25b and 25c are 20 times more abundant in testis than transcripts initiating upstream of exon 25 (Fig. S5A). This strongly suggests that the vast majority of *Tln2* transcripts in testis initiate downstream of exon 25. 5′-RACE experiments confirmed that exon 25b represents a genuine *Tln2* transcription start site in testis. The position of the start site varies slightly in the three clones that we sequenced, and no TATA-box was detected upstream of exon 25b (Fig. 2B). From the above results, we conclude that these short *Tln2* transcripts are expressed at high levels specifically in testis, and that they contain a unique 5′-end composed of either exon 25b or exons 25b and 25c.

To establish whether the ∼ 4.8 kb *Tln2* transcripts in testis gives rise to truncated talin 2 protein(s), we used a gene trap mouse line [24] in which the transgene is inserted between exons 28 and 29 (Fig. 2A). Because the gene trap cassette is downstream of exons 25b and 25c, it is predicted to interrupt the transcription of both long (ubiquitous) and short (testis-specific) *Tln2* transcripts. If full-length talin 2 (270 kDa) and the shorter isoforms (153 kDa) coexist in testis, they should both be detected in wild-type animals with an antibody raised against the C-terminal region of talin 2, whereas both proteins should be absent in homozygous gene trap mice. Furthermore, the gene trap cassette should lead to the expression of two talin 2–βGeo fusion proteins that can be detected by an antibody against β-galactosidase (Fig. 2D). As predicted, the levels of talin 2 proteins were dramatically reduced in tissues from homozygous (gt/gt) mice, and they were replaced by the predicted talin 2–βGeo fusions (Fig. 2D). These results establish for the first time the existence of a truncated talin 2 in testis, which we have named Ts-talin 2 (testis-talin 2). We have not detected Ts-talin 2 in any other tissues.

As Ts-talin 2 lacks the integrin-binding site found in the N-terminal FERM domain of full-length talin, we sought to establish whether it could localize to cell–ECM junctions. Analysis of NIH-3T3 cells transiently expressing green fluorescent protein (GFP)–Ts-talin 2 confirmed that the protein was efficiently expressed (Fig. S6A and Doc. S1), and that it localized to focal adhesions in a similar manner to GFP–full-length talin 2 (Fig. S6B and Doc. S1), mCherry–full-length talin 2 or endogenous full-length talin 2 [25]. Expression of GFP–Ts-talin 2 in talin 1-deficient dJ26.28 cells [15,34] produced essentially similar results, showing that its localization is not dependent upon talin 1 (data not shown).

**Fig. 3:**
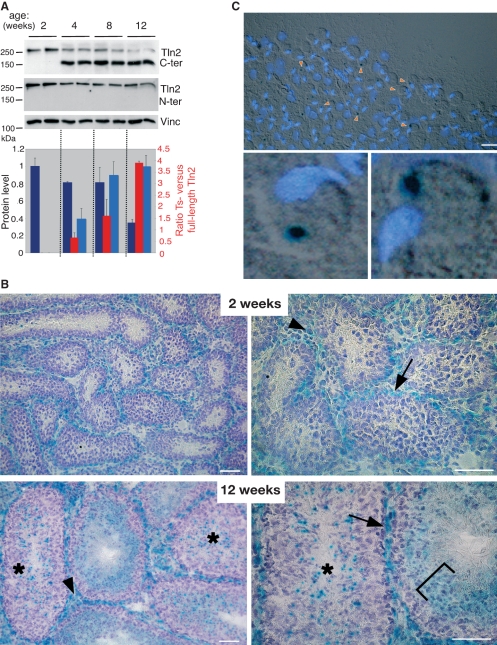
Ts-talin 2 is developmentally regulated and is expressed in early elongating spermatids. (A) Western blotting of testis protein extracts from two C57Bl/6 mice prior to (2 weeks old) and after puberty (4, 8 and 12 weeks). A talin 2 monoclonal C-terminal antibody was used to detect both full-length talin 2 and the short Ts-talin 2 isoforms, whereas the N-terminal monoclonal antibody detected only full-length talin 2. The respective levels of Ts-talin 2 (light blue), full-length talin 2 (dark blue) and the Ts-/full-length talin 2 ratio (red) were assessed by luminometry (average of two animals per age group; relative to vinculin loading control). (B) 5-bromo-4-chloro-3-indolyl-β-d-galactopyranoside (X-gal) staining of cryosections of 2-week-old and 12-week-old testis from animals with a gene trap insertion reporting the expression of both full-length and Ts-talin 2 (see Fig. 2). Arrows indicate expression in myoid cells adjacent to the basement membrane of the seminiferous tubule; arrowheads point to the staining of interstitial Leydig cells. Staining in both locations was identical in prepubertal and pubertal mice. An asterisk shows the punctate expression detected only after puberty, restricted to specific stages of the tubule cycle and replaced by a diffuse staining of the adluminal compartment at the point of spermiation (bracket). Left panel: 20× objective. Right panel: 40× objective. Bar = 50 μm. (C) Differential interference contrast image of a stage XII–I seminiferous tubule squash (top panel) from a 3-month-old gene trap animal, stained with DAPI and X-gal, and detail of two elongating spermatids (bottom panel). Arrowheads indicate β-galactosidase staining. The data show that the punctate talin 2 expression in the mature testis, most likely Ts-talin 2, is restricted to the cytoplasm of elongating spermatids. Bar = 20 μm.

In order to further understand the role of Ts-talin 2 in testis physiology, we performed western blot analysis of testis extracts from prepubertal and pubertal mice using N-terminal and C-terminal antibodies to detect the full-length and short testis isoforms. The results show that Ts-talin 2 is not expressed in prepubertal testis (2 weeks) but is upregulated at the onset of puberty (4–6 weeks in the C57Bl/6 mouse strain). Concomitantly, the full-length protein is downregulated (Fig. 3A). In addition, our gene trap mouse line, which reports expression of both full-length talin 2 and Ts-talin 2 (Fig. 2A), allowed us to follow the expression pattern of the two proteins during this developmental switch. In heterozygotes, the β-galactosidase fusion protein was expressed in myoid cells and Leydig cells at the periphery of the seminiferous tubules, and this pattern of expression was identical at 2 and 12 weeks of age. However, strong punctate β-galactosidase staining appeared in the seminiferous epithelium after puberty. This was detected in only a subset of immature tubules, and was replaced by more diffuse staining as the tubule progressed towards the spermiation point (Fig. 3B). Dissection of portions of seminiferous tubules, staged by transillumination [35], revealed that these dot-like structures are only present between the end of stage VIII (end of spermiation), when the transition between round and elongating spermatids is initiated, and the end of stage I, when elongating spermatids start forming bundles [36] (data not shown). Squashes of tubules from stages VIII–XII and XII–I [35] were used to analyse further the expression of the β-galactosidase–talin 2 fusion protein. This showed that the dot-like structures are localized in the cytoplasm of early elongating spermatids (Fig. 3C). A proportion of dots were also observed in enucleated vesicles (data not shown), reminiscent of the residual bodies lost during the process of spermatid elongation [37]. Together with our western blotting data, these results strongly suggest that the expression of full-length talin 2 is restricted to myoid and Leydig cells, whereas Ts-talin 2 is characteristic of the early stages of spermatid elongation.

### A second, shorter *Tln2* transcript is highly expressed in kidney

**Fig. 4:**
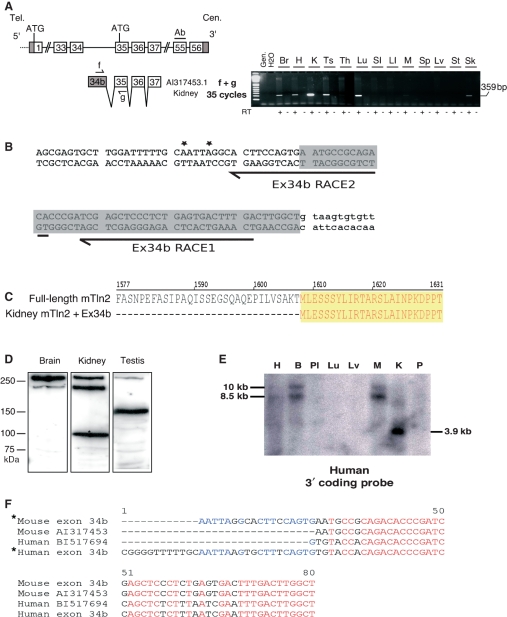
Evidence for a truncated mouse *Tln2* transcript and protein product highly expressed in kidney and conserved in human. (A) Schematic diagram of the mouse *Tln2* genomic region between coding exons 33 and 56. An EST containing the additional exon 34b is shown below. Grey boxes represent noncoding exons; white boxes are coding sequences. Half-arrows indicate the primers used in RT-PCR. RT-PCR amplification after 35 cycles using primers *f* + *g* is shown. Gen., genomic DNA; H_2_O, negative water control; RT+, reversed-transcribed RNA; RT−, non-reversed-transcribed control. Br, brain; H, heart; K, kidney; Ts, testis; Th, thymus; Lu, lung; SI, small intestine; LI, large intestine; M, skeletal muscle; Sp, spleen; Lv, liver; St, stomach; Sk, skin. (B) 5′-RACE confirms the 5′-end of the short transcript. The sequence of exon 34b is shown, and the position of the EST is shaded grey; intronic sequences are in lower case; the position of the RACE primers is indicated by an arrow, and the positions of the 5′-ends of two sequenced RACE amplicons are marked by a star. (C) Alignment of the predicted N-terminus of the corresponding truncated protein with the full-length talin 2. (D) Western blotting showing the presence of a 90 kDa talin 2 protein in kidney (*). (E) Conservation of the short ‘kidney’ transcript in humans. A human tissue northern blot was probed with a DNA fragment detecting the 3′ coding region of *TLN2*. A 3.9 kb transcript strongly expressed in kidney was detected. (F) A kidney cDNA library was screened with primers near the putative 5′-end of the transcript and in the cloning vector. This revealed a new exon that was also present in a human EST. The alignment of the corresponding cDNA sequences with the equivalent mouse sequences is shown. *, 5′-ends determined in this study.

Northern blotting detects a highly expressed 3.9 kb *Tln2* transcript in kidney [12,24]. Examination of mouse ESTs revealed a kidney EST (AI317453) with a novel 5′-exon (numbered 34b) just downstream of exon 34 (Fig. 4A and Table S2). The position of exon 34b suggests that it may represent the first (or one of the first) exon(s) of the 3.9 kb *Tln2* kidney transcript (Fig. 4A). RT-PCR showed that exon 34b-containing transcripts are expressed at very high levels in kidney and at lower levels in brain, heart, testis, lung, muscle, and skin (Fig. 4B). Moreover, quantitative real-time RT-PCR showed that exon 34b transcripts are 11 times more abundant in kidney than those initiating upstream (Fig. S5B). Conversely, in small intestine and brain, exon 34b transcripts are expressed at much lower levels than the full-length transcripts (Fig. S5B,C). Together, these results indicate that exon 34b is part of a different *Tln2* transcript that is mainly expressed in kidney, where it is by far the most abundant species.

To confirm that exon 34b is actually the first exon of the 3.9 kb kidney *Tln2* transcript, we performed 5′-RACE. The data show that transcripts containing exon 34b do not carry any further 5′-exons (Fig. 4B). The longest 5′-RACE product that we have identified does not contain any ATGs in exon 34b that are in-frame with the rest of talin 2, so it is very likely that exon 34b is a noncoding exon. The first ATG in-frame with the *Tln2* ORF is found in exon 35, which is common to both long and short transcripts (Fig. 4A,C). The corresponding truncated talin 2 isoform is predicted to be a 90 kDa protein spanning residues 1608–2543 (Figs 4C and 6). This isoform should not be detected by antibodies raised against the N-terminal region of talin 2, whereas an antibody against the C-terminal region should detect both full-length talin 2 and an additional 90 kDa protein, mainly in kidney. As expected, western blotting with the N-terminal antibody showed only full-length talin 2 (data not shown). In contrast, two different C-terminal antibodies detected an additional protein of ∼ 90 kDa that was strongly expressed in kidney (Fig. 4C and data not shown).

To investigate whether a similar talin 2 isoform is expressed in humans, we first probed a northern blot using a conserved mouse *Tln2* 3′-probe. As in mouse, we observed a 3.9 kb transcript that was specifically and highly expressed in human kidney (Fig. 4D). In addition, a search for sequences homologous to exon 34b in the EST database revealed the existence of a human kidney EST (BI517694) containing at its 5′-end the conserved counterpart of exon 34b. By screening a human kidney cDNA library with a set of nested primers in the conserved exon 34b and a second primer in the vector (Sp6), we were able to amplify a 450 bp cDNA product, which was cloned and sequenced. Alignment between the new human sequence, the mouse 5′-RACE product and the mouse and human ESTs containing exon 34b revealed that exon 34b is highly conserved between the two species, and does not have any coding potential (Fig. 4D). We conclude that the shorter 3.9 kb transcript and exon 34b are conserved in humans, and, as in mice, are highly expressed in kidney.

### Alternative splicing of *Tln2* coding exons

**Fig. 5:**
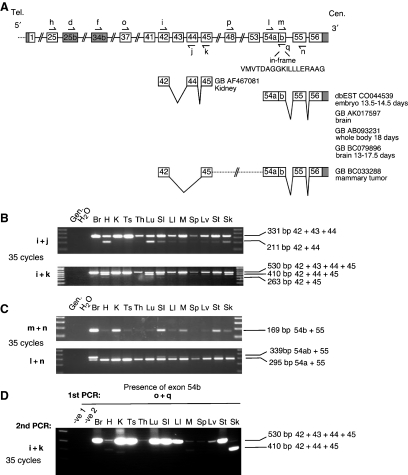
Evidence for alternatively spliced m*Tln2* transcripts. (A) Schematic diagram of two regions showing alternative splicing. ESTs supporting alternative splicing are shown below the genomic region. Primers used to detect alternative splicing (*i*, *j*, *k*, *l*, *m* and *n*) and short transcript isoforms (*d*, short testis transcript; *f*, short kidney transcript) are indicated by half-arrows. The amino acid sequence of the in-frame peptide added as a result of inclusion of exon 54b is shown. (B, C) RT-PCR amplification after 35 cycles using various primer combinations. (B) Primer *i* + *j* detects splicing out of exon 43; *i* + *k* reveals splicing of both exon 43 and exon 44. (C) *m* + *n* indicates the presence of exon 54b; *l* + *n* indicates the relative abundance of exon 54a + exon55 and exon 54ab + exon 55 products. Gen., genomic DNA; H_2_O, negative water control; RT+, reversed-transcribed RNA; RT−, non-reversed-transcribed control. Br, brain; H, heart; K, kidney; Ts, testis; Th, thymus; Lu, lung; SI, small intestine; LI, large intestine; M, skeletal muscle; Sp, spleen; Lv, liver; St, stomach; Sk, skin.

The existence of alternatively spliced transcripts has been described in vertebrate *talin 2* and in the unique ancestral *talin* gene, which is closely related to *talin 2* [12,13]. In order to clarify the extent of alternative splicing in the mammalian gene, we conducted a systematic review of alternatively spliced ESTs in the mouse. We also sought to determine the expression profile of these variants as well as their relationship to the various short transcripts described above. We identified three genuine *Tln2* splice variants in mouse ESTs (Fig. 5A). The first alternative splicing skips exon 43 (kidney AF467081). The second one, which contains an extension of exon 54 (exon 54b; Table S2) and inserts 15 amino acids between residues 2458 and 2459 of the protein, is found in four distinct ESTs (brain AB093231, CO044539 and BC079896; 8 days *post coitum* whole body AK017597), and is equivalent to a splice variant previously described in humans [13]. The third variant EST from a mammary tumour library combines skipping of exons 43 and 44 with the inclusion of exon 54b (BC033288).

RT-PCR showed that exon 43 skipping is common in most adult tissues (Fig. 5B; primers *i* + *j* and *i* + *k*), although the ratio between transcripts including or excluding exon 43 varies greatly between tissues. Only heart, lung and skin express the exon 43-minus variant at about the same level as the wild-type transcript, whereas it is found at much lower levels in other tissues, and was totally absent from brain. In contrast, skipping of exons 43 and 44, as found in a mammary tumour EST, is a rare event in the tissues tested (Fig. 5B; primers *i* + *k*). Next, we searched for transcripts that included exon 54b. Again, this splice variant was detected in all the tissues tested, but at variable levels (Fig. 5C; primers *m* + *n*). Strikingly, transcripts containing exon 54b are the major variant in brain (Fig. 5C; primers *l* + *n*).

To search for mRNAs lacking exons 43 and 44 but including exon 54b, we first amplified cDNAs containing exon 54b (primers *o* + *q*), and then assessed the splicing of either exon 43 alone or exons 43 and 44 using nested PCR (primers *i* + *k*; Fig. 5D). We did not detect any products carrying deletions of both exons 43 and 44 (Fig. 5D), which confirmed the very low levels previously observed in the total cDNA population (see above and Fig. 5B). However, transcripts skipping exon 43 and containing exon 54b are a major component of skin, whereas in kidney and muscle they occur at a much lower level. We also failed to detect exon 43 skipping in either the testis or kidney short transcripts (Fig. S7), which suggests that skipping of exon 43 is a specific feature of long transcripts. In contrast, inclusion of exon 54b was found in both the short and long transcripts expressed in brain and kidney, but not in heart or testis (Fig. S7). We conclude that the short *Ts-Tln2* transcripts do not skip exon 43 or include exon 54b, whereas in kidney, exon 54b is mainly added to the short transcript, and is much less abundant in full-length *Tln2* transcripts. Together, these results provide new evidence of the complexity of *Tln2* transcription and suggest that the gene encodes at least three different transcripts, which are subject to alternative splicing in a tissue-specific and/or isoform-specific manner.

## Discussion

In the present study, we have carried out a detailed analysis of mouse *Tln2* and showed that it spans over 400 kb. This is much larger than previously reported, owing to the presence of a new promoter associated with a CpG island located 236 kb upstream of the first coding exon. This promoter drives transcription of *Tln2* mRNAs containing a number of 5′-exons scattered over ∼ 200 kb, none of which generate an ORF in-frame with the rest of the *Tln*2 coding sequence. We conclude that they are 5′-UTR exons. These untranslated exons are subject to alternative splicing, and this probably explains why several large *Tln2* transcripts can be detected in northern blots [12,24]. The smallest RT-PCR amplicon derived from the mouse 5′-UTR is composed of exons −7, −5 and −2. When this is linked to the sequence encoding the full-length protein, it gives rise to a 9.2 kb transcript, whereas a *Tln2* transcript containing all of the authenticated 5′-UTR exons (−7, −6, −5, −2 and 0) would be about 10 kb in length. These are similar in size to the 9.5 kb and 10 kb transcripts detected in northern blots [12,24]. However, we have yet to determine the full extent of alternative splicing of the 5′-UTR exons. The failure to detect transcripts containing exons −4 and −1 in adult mouse tissues (Fig. S1) suggests that their inclusion is tissue-specific and/or developmentally regulated. It also raises the question as to whether they are ever included in *Tln2* transcripts initiating at the CpG island. Interestingly, exons −4 and −1 are the most 5′ sequences in ESTs derived from mouse blastocysts and 13-day-old embryo heart respectively (Fig. 1A), suggesting that there may be alternative transcriptional start sites in the vicinity of exons −4 and −1.

With respect to human *TLN2*, previous studies have assumed that transcription is initiated at the first base of the first coding exon [25]. However, we demonstrate that some of the 5′-UTR exons observed in the mouse are conserved in human *TLN2*, and that large *TLN2* transcripts exist in human tissues. Moreover, using RT-PCR and human fibroblast and heart mRNAs, we detected additional novel 5′-UTR exons linking exon −7 to exon 1. Thus, the 5′-UTR of human *TLN2* is similarly complex, although not all the 5′-UTR exons are conserved between human and mouse (Fig. S2). The role of such a complex alternatively spliced 5′-UTR is unclear, although its conservation between species implies an important functional role. It could, for instance, regulate the stability of the corresponding transcripts at different levels, either intrinsically or in response to cellular cues [38,39].

Our data in mouse show that a major *Tln2* promoter is associated with the CpG island upstream of these 5′-UTR exons, and that this is conserved in all mammals. The *in silico* characterization of this promoter was based on the rationale that polymerase II promoters usually contain multiple TF-binding sites, the spatial organization (framework) of which defines the specificity of the promoter [28,29]. Using this stringent approach, we identified a minimal core promoter that is conserved in mammals and contains a common framework of binding sites for the ubiquitously expressed TFs nuclear respiratory factor 1, E2F, cAMP-responsive element binding protein and ETS-1. However, of the above TFs, only ETS-1 has previously been implicated in cell adhesion, via transcriptional regulation of metalloproteases [40]. Clearly, further experimental work will be required to confirm the role of these TFs in regulating *Tln2* expression. Interestingly, in humans, the region that we have defined as the conserved minimal promoter is the only *TLN2* sequence bearing chromatin features characteristic of a promoter [41] (Fig. S4).

Direct experimental proof that the distal CpG region contains a major *Tln2* promoter comes from studies with mice carrying a gene trap insertion in intron −3. The results establish that the majority of talin 2 expressed in adult tissues is derived from transcripts that initiate upstream of exon −3. However, the gene trap insertion failed to totally abolish talin 2 expression, and the levels of residual talin 2 varied between tissues. This may be due to splicing around the gene trap, the efficiency of which may vary from tissue to tissue, or to the presence of a tissue-specific promoter(s) downstream of the insertion. Indeed, the existence of a muscle-specific promoter in human *TLN2* just upstream of the ATG of exon 1 has been reported [25], although the authors only identified individual TF-binding sites *in silico* rather than conserved clusters. If this promoter is authentic, then our data show that it is clearly not the only promoter used in skeletal muscle (Figs 1B, 4D and S2B).

**Fig. 6:**
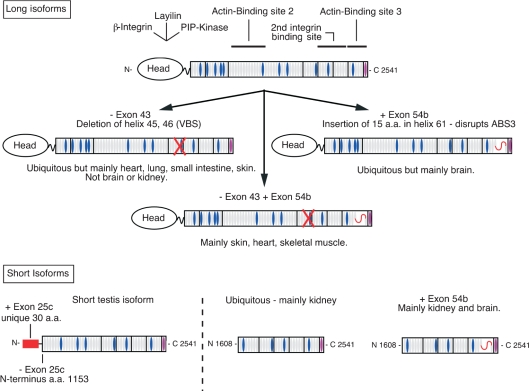
Diagram of the main talin 2 isoforms. Top: model of full-length talin 2 based on the assumption that the main structural features and ligand-binding sites of talin 1 are conserved in talin 2. Vinculin-binding sites (VBS) are shown in blue. The consequences of the three main alternative splicing events detected by RT-PCR for the structure of the long talin 2 isoforms (Figs 5 and S7) are indicated. A red cross indicates that exon 43 skipping (−exon 43) deletes VBS3. A red curved line shows the 15 amino acids inserted into the C-terminal actin-binding site (ABS3) by the inclusion of exon 54b (+exon 54b). Bottom: structures of the main short talin 2 isoforms predicted to arise from smaller *Tln2* transcripts.

The next level of complexity resides in the existence of additional *Tln2* promoters that give rise to short *Tln2* transcripts in certain tissues, the evidence for which was originally based on northern blots of testis and kidney [12]. We have verified the existence of two short *Tln2* transcripts that are restricted to adult testis, and that differ at the 5′-end, owing to alternative splicing of the first coding exon (exon 25c). This should result in the expression of two short talin 2 isoforms that span residues 1121–2542 and 1153–2542 of the full-length talin 2, the notable difference being that the former contains a unique 30 amino acid N-terminal extension encoded by exon 25c (Figs 2 and 6). We have confirmed by western blotting that the predominant talin 2 isoform in testis has a molecular mass of ∼ 150 kDa and that its expression correlates with the onset of puberty in mice. Furthermore, analysis of the β-galactosidase–talin 2 reporter generated by the insertion of a gene trap downstream of the testis-specific transcriptional start site (intron 28; see Fig. 2) revealed that upregulation of Ts-talin 2 at puberty correlates with the appearance of strong and localized X-gal staining in the cytoplasm of early elongating spermatids and in enucleated vesicles, reminiscent of residual/cytoplasmic bodies [37]. Therefore, we propose that Ts-talin 2 might play a key role in the regulation of spermatid elongation and/or migration through the seminiferous epithelium. These processes are thought to involve cell–cell contacts between elongating spermatids and the apical ectoplasmic specializations (ESs) of surrounding Sertoli cells. Various laminin isoforms, integrin α6β1 heterodimers, F-actin and other cytoskeletal and signalling molecules have been implicated on both sides of the spermatid–ES junction, although, interestingly, the linker proteins between the extracellular laminins and the cytoplasm of the elongating spermatid are currently unknown [42,43]. If Ts-talin 2 is important in spermatogenesis, one would predict that spermatogenesis should be defective in mice homozygous for the gene trap insertion in intron 28, which results in a dramatic reduction in the level of Ts-talin 2. However, these mice are viable and fertile [24]. Quantitative RT-PCR across the insertion site, and careful examination of western blots, reveals that transcription downstream of the gene trap still occurs, and a low level of the Ts-talin 2 is still present in the testis of these mice (data not shown). Thus, the gene trap may well be a hypomorphic rather than a null allele, and a more thorough investigation of the phenotype of these mice is required.

The second short *Tln2* transcript identified here contains a unique 5′ noncoding exon (34b), and was found mainly but not exclusively in kidney. Using western blotting, we have confirmed that the corresponding 90 kDa protein is expressed in kidney, although we have not detected it in embryonic or adult kidney epithelial cell lines (NRK, HEK-293, Cos-7; data not shown) or in other tissues. However, the expression of the corresponding short transcripts in other tissues suggests that the protein(s) may be present at low levels, perhaps in a subset of cells. The protein is predicted to span residues 1608–2542, and therefore lacks the N-terminal region of full-length talin 2 (Fig. 6).

Recent studies show that the N-terminal FERM domain of talin 1 is important in both integrin activation [44,45] and in the intramolecular head–rod interaction that is thought to keep cytoplasmic talin 1 in the autoinhibited form, in which the integrin-binding sites are masked [46] (B. T. Goult, N. Bate, N. J. Anthis, K. L. Wegener, A. R. Gingras, B. Patel, I. L. Barsukov, I. D. Campbell, G. C. K. Roberts & D. R. Critchley, unpublished results). Clearly, these regulatory functions have been lost from both the testis and kidney short talin 2 isoforms, and in testis, these may not be required, given the unique architecture and molecular composition of the ES junctions [42,43]. However, both isoforms contain the second integrin-binding site in the rod as well as the highly conserved C-terminal actin-binding site and several vinculin-binding sites (Fig. 6), and therefore still retain the potential to link integrins to the actin cytoskeleton.

Evidence has recently come to light regarding the existence of a microRNA, *miR-190*, in sense orientation within intron 51 of mouse *Tln2* (ensembl, genome release 50, gene ENSMUSG00000076379, coordinates chromosome 9: 67 084 458–67 084 542). *MiR-190* is highly conserved both in sequence and in position in *talin 2* in all vertebrates analysed so far. It is also present and expressed from the unique *talin* gene of insects (12 *Drosophila* species [47] and the honey bee, *Apis mellifera* [48]), thus confirming the relationship between the ancestral *talin* gene and *talin 2* [13]. However, *miR-190* is absent in the *Caenorhabditis elegans* genome, indicating that *miR-190* first appeared in the Arthropod lineage and was maintained throughout the chordates. Interestingly, *miR-190* is expressed in mouse testis and kidney [49,50], where the two *Tln2* short transcripts are highly expressed, as well as in various human cell lines [50], and is upregulated in primary myelofibrosis patients [51]. As *miR-190*, like many intronic microRNAs [52], is transcribed as part of larger primary transcripts, it is possible that one of the functions of the short *Tln2* transcripts is to produce *miR-190*. Intriguingly, out of the 36 *miR-190* target genes common to at least two different prediction algorithms and conserved in mammals [51], 12 are genes involved in cell adhesion or related processes, e.g. members of the ARP 2/3 complex (*ACTR3* and *ARPC5*), cadherins (*CDH2*, *PCDH9*), myosin VA (*MYO5A*), dystroglycan and attractin precursors (*DAG1* and *ATRN*), neurofascin (*NFASC*), hyalouronan synthase (*HAS2*), and *KRAS2*. Clearly, it will be important to establish the origin of *miR-190* transcripts, and their expression profile, targets, and function.

In addition to the short talin isoforms, we have also identified four alternatively spliced *talin 2* transcripts. Exon 43 is skipped in one variant, and comparison of talin 1 and talin 2 sequences (74% identity) shows that this would delete residues 1912–1960, equivalent to helix 45 and helix 46 of the talin 1 rod (Protein Data Bank ID: 2b0h). Helix 46 contains one of the numerous vinculin-binding sites (VBS3; Fig. 6) in talin [10], and deletion of this region would be predicted to destroy this site. Unfortunately, it has not been possible to detect the corresponding protein by western blotting, because the difference in molecular mass (5.46 kDa) does not allow separation from full-length talin 2. We have also detected inclusion of exon 54b in the main *talin 2* transcript expressed in brain and at lower levels in other tissues. The extra 15 amino acids encoded by exon 54b, which are totally conserved between mouse and human [13], are inserted into the N-terminal region of helix 61 of the talin rod. In talin 1, this helix is part of a five-helix bundle that forms the C-terminal actin-binding site [53], and therefore the insertion could have important functional implications (Fig. 6). Clearly, an antibody against the extra amino acid sequence will be required to establish whether the corresponding protein is actually expressed in brain.

In summary, our results demonstrate the complexity of the mammalian *talin 2* gene and provide direct evidence for the existence of at least three different protein isoforms expressed in a tissue-specific manner. Moreover, the occurrence of four alternative splicing events raises the possibility that the number of talin 2 isoforms is actually much higher. Thus, we currently predict the existence of six talin 2 variants in addition to the three short protein isoforms (Fig. 6). This highlights the need to use isoform-specific talin 2 antibodies, where possible, for immunolocalization and biochemical studies. The results also have important implications for the design and interpretation of experiments aimed at defining the expression and the function of talin 2. First, as there are several *Tln2* promoters, the use of gene traps as reporters of *Tln2* expression patterns should be considered with care. Second, the fact that the *Tln2* gene traps fail to ablate talin 2 expression indicates that exon skipping may cause problems in designing *Tln2* mutant alleles. Third, the use of small interfering RNAs downstream of exon 25b or exon 34b should be avoided, as they will affect expression of both full-length and the shorter variant(s) of talin 2. Instead, targeting of transcript-specific exons (e.g. 25b, 25c, or 34b) and/or promoters might be preferrable. Fourth, the presence of microRNA *miR-190* within the 3′-region of the *Tln2*/*TLN2* gene raises questions about its function, the role of the short *Tln2* transcripts, and the protein isoforms that they encode, and should also be taken into consideration in the design of knockout strategies.

## Experimental procedures

### EST and cDNA analysis

We used the National Institute for Allergy Mouse Gene Index (version 5) and the Mouse Genome Assembly through the ensembl portal (build 37; NCBI reference sequence version 36) to carry out our analysis. The National Institute for Allergy Mouse Gene Index gene index provided a graphical view of the *Tln2* structure, a list of all the known and predicted *Tln2* exons, and individual splicing events along with a list of supporting ESTs and cDNAs. This information was analysed manually and compared with the data present in ensembl. Together, this provided a framework with which to decipher *Tln2* structure, the existence of short transcripts, and the presence of alternative splicing events. It was also used to design the various primers relevant to the characterization of the gene structure and described in Table S1.

### Promoter prediction

blast was used to identify the sequences equivalent to the most 5′ mouse *Tln2* exon (exon −7) in human, chimpanzee, mouse, rat, dog and cow genomic sequences. A 2.2 kb sequence including the 5′-end of the CpG island was extracted from each individual genomic sequence via ensembl, and analysed using the genomatix promoter analysis suite (http://www.genomatix.de). Briefly, we used frameworker and the most stringent settings to identify the most complex model (framework) of TF-binding sites that occurred in the same order and in a similar region and that were common to all six sequences. In addition, dialign tf [54] was used to identify TF-binding site matches within the multiple sequence alignments using default parameters. TF-binding site matches were identified by comparison with a matrix of descriptions with matinspector. To minimize artefacts due to the small size of TF-binding sites, we increased the threshold of similarity to the matrix to 0.99, and identified only TF-binding site matches that were common to all six sequences.

### RNA extraction

Tissues from wild-type C57/Bl6 mice were dissected and stored in RNAlater (Invitrogen, Carlsbad, CA, USA), and total RNA was extracted from a 20 mg piece of tissue using the RNAeasy Miniprep kit (Invitrogen), both according to the manufacturer’s instructions. RNA concentrations were quantified at 260/280 nm and treated accordingly with RQ1 DNase I (Promega, Madison, WI, USA; 1 unit·μg^−1^ RNA). After addition of 600 μL of RLT buffer (Invitrogen; RNAeasy miniprep kit), samples were purified through a second RNA column (Invitrogen; RNAeasy miniprep kit) to eliminate the enzyme. Yields were quantified at 260/280 nm prior to storage at −80 °C.

### Human RNA, tissue northern blot and kidney cDNA library

Human foreskin fibroblast and adult heart total RNAs were a kind gift from K. Clark (University of Leicester, UK); heart RNA was from Stratagene (Santa Clara, CA, USA; #540011). One microgram of RNA was reversed transcribed using a standard protocol (see below). A human Multiple Tissue Northern Blot was purchased from BD Biosciences Clontech (Palo Alto, CA, USA) and hybridized as previously described [12] with a 598 bp mouse cDNA probe spanning exons 52–55. In order to map the 5′-end of the short *Tln2* transcript present in human kidney, plasmid DNA from a human kidney cDNA library (kindly provided by M. J. A. Tanner, University of Bristol) was screened by PCR. We took advantage of the directional cloning of the cDNAs in the vector, and primer SP6-2 in the plasmid sequence flanking the 5′-end of the cDNA inserts was used in combination with primer hT2-3R, designed in reverse orientation in the *TLN2* sequence near the putative 5′-end of the short transcript (Table S1). A 450 bp amplicon was cloned and sequenced.

### Reverse transcription

DNAse-treated total RNA (1 μg) was reversed transcribed with Superscript III (Invitrogen) and random primers (Promega) as recommended by Invitrogen, with 250 ng of random primers in a total volume of 20 μL. In addition, non-reversed transcribed controls were generated in the same conditions by replacing the Superscript III enzyme with water. One to two microlitres of these reactions was used in PCRs with the primers described in Table S1. All primers used to detect cDNA were designed to span at least one exon–intron boundary, to avoid detection of residual genomic DNA.

### PCR

The amplification of small amplicons (< 2 kb) was performed in standard conditions at an annealing temperature of 55 °C, using 600 nm of the primers described in Table S1 and PCR Reddy Mix (ThermoScientific, Walthman, MA, USA) in a volume of 15 μL. When a second round of nested PCR was required, 1 μL of the first round was used in a 15 μL reaction as described above. Long-range PCR (> 2 kb) was performed using the Expand High Fidelity PCR kit (Roche, Indianapolis, IN, USA), according to the manufacturer’s recommendations, with 300 nm of each primer. One microlitre of these reactions was used subsequently as template in a second nested PCR with ReddyMix as described above.

### Real-time quantitative RT-PCR

Expression of *Tln2* variant mRNAs was quantified using real-time PCR in a BioRad MiniOpticon Thermocycler with 2x iQ SyberGreen Mastermix (BioRad, Hercules, CA, USA) and the specific primer sets described in Table S1 and in the figure legends. Amplification conditions, annealing temperature and primer pair efficiency (95–105%), were optimized for each primer pair prior to the experiment, to validate the comparison between different primer pairs. Primers were used at a final concentration of 300 nm in a 25 μL reaction volume. One microlitre of random-primed cDNA from relevant tissues was added to each reaction, and each cDNA sample was amplified in triplicate to take into account variations in amplification. *Gapdh* was used as an internal normalization control. Relative abundances were calculated using the 2^−ΔΔCt^ method [55].

### Gene trap ES cells

The gene trap ES cell line RRI4343 (plasmid insertion) was obtained from BayGenomics (Stamford, CA, USA), and the FHCRC-S1-D6 gene trap (lentiviral insertion) from P. Soriano’s laboratory (Seattle, WA, USA). These ES cell lines were grown in DMEM with 4.5 g·L^−1^ glucose plus glutamine and 10% fetal bovine serum on 0.1% gelatin (RRI4343) or mitomycin C-treated MEF feeder layers (FHCRC-S1-D6) in the presence of 1000 U·mL^−1^ leukaemia inhibitory factor, as described previously [19]. The clones were expanded and stained with X-gal to check for β-galactosidase expression, and genomic DNA was extracted and amplified by PCR to verify the presence of the correct gene trap insertion. The insertion point of the gene trap construct in cell line RRI434 was determined by Chen & Lo [24]. We used this information to design our own set of genotyping primers, 5′GT4/GT4Vec (gene trap allele, 450 bp) and 5′GT4/3′GT4 (wild-type allele, 700 bp; for sequences, see Table S1), which were used in a single multiplex PCR. The genomic region upstream of the insertion point of the lentivirus in clone S1-D6 was cloned and sequenced by P. Soriano’s team, and the sequence information provided was used to design primer pairs SGT5′For/SGT5′Rev (gene trap allele, 684 bp) and SGT5′For/SGT3′Rev (wild-type allele, 536 bp; see sequences in Table S1), which were used in separate standard PCR reactions.

### Generation of transgenic mice and genotyping

ES cell clones RRI434 and FHRC-S1-D6 were injected into C57BL/6 blastocysts. Germline transmission from male chimeras was assessed through the presence of *Agouti* pups in the offspring. Animals carrying the gene trap insertions were identified by PCR using genomic DNA extracted from ear snips, using the HotSHOT technique as previously described [56]. Heterozygous gene trap mice (gt/+) were either outbred onto a C57/Bl6 background to establish a colony or intercrossed in order to generate homozygotes (gt/gt). Combinations of primers (see gene trap ES cells and Table S1), detecting both the presence and the absence of the gene trap at the insertion point, allowed discrimination between heterozygous and homozygous mice.

### Protein lysates from tissues and western blotting

Tissues were dissected from genotyped animals, flash frozen in liquid nitrogen, and stored at −80 °C. The frozen tissues were lysed and homogenized with a Polytron tissue homogenizer as described in [57], in the presence of a cocktail of protease inhibitors (Calbiochem, San Diego, CA, USA; Set III; 1 : 100 dilution) plus a calpain II inhibitor (E64d; Sigma, St Louis, MO, USA; 1 μg·mL^−1^). Protein concentrations were determined in 5 μL aliquots in triplicate using the Bradford assay (Coomassie Plus Kit; ThermoScientific) with BSA standards. Aliquots of tissue lysates containing 40 μg of protein were denatured at 100 °C for 2 min in the presence of 5 mm Tris/HCl (pH 6.8), 4% glycerol, 1.6% SDS, 2% bromophenol blue, and 1%β-mercaptoethanol. The samples were subjected to SDS/PAGE (6% gels), and the proteins were electroblotted onto Hybond P membranes using wet transfer in 2.5 mm Tris base and 9.6 mm glycine. Excess protein binding sites were blocked by incubation in 5% dried milk in NaCl/Tris (pH 7.4). The long isoform of talin 2 was detected using a mouse monoclonal antibody (68E7) raised in this laboratory against an N-terminal recombinant mouse talin 2 peptide (amino acids 482–911) and diluted 1 : 200 in blocking buffer. Polypeptides containing the C-terminal region of talin 2 were detected with an in-house mouse monoclonal antibody (53-121A1, epitope 2479-2494; 1 : 50 dilution) or a polyclonal antibody raised in rabbits against residues 2477–2491 of human talin 2 (1 : 3500 dilution). Vinculin was detected using the mouse monoclonal antibody F9 as previously described, with a 1 : 400 dilution [13]. β-Galactosidase was detected with a rabbit polyclonal primary antibody (Abcam, Cambridge, UK; ab4761, 1 : 1000 dilution). Bound primary antibodies were detected by adding anti-mouse IgGs (GE Healthcare UK Ltd, Chalfont St Giles, UK; 1 : 2500 dilution) or anti-rabbit IgGs (Sigma; A6154, 1 : 3500) coupled to horseradish peroxidase. Horseradish peroxidase activity was detected using an ECL Kit (SuperSignal West Pico; ThermoScientific), and autoradiographic films were exposed for varying amounts of time. Semiquantitation of the relative abundance of talin 2 across samples was performed using Adobe photoshop. Films were scanned and the images were inverted. Rectangles of constant surface area were drawn around the talin 2 and vinculin bands, and the mean luminosity of each rectangle was determined in Adobe photoshop. Levels of talin 2 were obtained by normalizing the talin 2 to the vinculin signals and by comparing the values between samples.

### Cryosections and X-gal staining

Testes were dissected and frozen in optimal cutting temperature (OCT) medium (R. A. Lamb, London, UK) and 20 μm frozen sections were fixed in 2% paraformaldehyde and 0.1% glutaraldehyde for 5 min, and stained for β-galactosidase activity overnight at 37 °C in 1 × NaCl/P_i_ with 2.5 mm X-gal, 5 mm potassium ferrocyanide, 5 mm potassium ferricyanide, 2 mm MgCl_2_, 2% sodium deoxycholate and 0.4% NP-40. The slides were washed three times in 1 × NaCl/P_i_, and nuclei were counterstained with haematoxylin. After being rinsed in cold water, the slides were dehydrated in 70, 90 and 100% ethanol, postfixed twice in xylene, and mounted in DPX synthetic resin.

### Seminiferous tubule dissection and X-gal staining

The testis was decapsulated, and the tubules were staged and dissected as previously described [35]. The relevant portions of tubules were squashed on a slide using a coverslip, and flash frozen in liquid nitrogen before being fixed in 2% paraformaldehyde for 5 min. X-gal staining was performed as described for cryosections, and nuclei were counterstained with 4′,6-diamidino-2-phenylindole dihydrochloride (DAPI; Invitrogen) for 5 min at room temperature. Slides were subsequently washed three times in 1 × NaCl/P_i_ before mounting. Differential interference contrast and DAPI-stained images were acquired on a Leica DM5000B microscope fitted with a 40× objective and a Leica DFC420C imaging system.

## Abbreviations

DAPI: 4′,6-diamidino-2-phenylindole dihydrochloride
E2F: elongation factor 2
ECM: extracellular matrix
ES: ectoplasmic specialization
EST: expressed sequence tag
ETS: E-twenty-six
GFP: green fluorescent protein
TF: transcription factor
*Tln2*: mouse *talin 2* gene
*TLN2*: human *talin 2* gene
X-gal: 5-bromo-4-chloro-3-indolyl-β-d-galactopyranoside

## Supporting information

The following supplementary material is available:

**Fig. S1.** Evidence for alternative splicing of the 5′-UTR talin 2 exons.

**Fig. S2.** Evidence for the conservation of the 5′-UTR exons and CpG island in human TLN2.

**Fig. S3.** De.nition of a promoter associated with the Tln2 CpG island and conserved in mammals.

**Fig. S4.** Human ensembl contig viewer windows showing chromatin immunoprecipitation (ChIP) on chip results in the 5′-region of TLN2, with only one region (CpG island) having promoter features.

**Fig. S5.** Quanti.cation of talin 2 short transcript levels in various tissues.

**Fig. S6.** Ts-talin 2 colocalizes with full-length talin 2 in NIH3T3 cells.

**Fig. S7.** Evidence for alternative splicing in short mTln2 transcripts.

**Doc. S1.** Materials and methods appendices.

**Table S1.** List and sequences of primers used in this work.

**Table S2.** List and sequence of mouse Tln2 exons.

This supplementary material can be found in the online version of this article.
