# Supplementary material for: Talin 2 is a large and complex gene encoding multiple transcripts and protein isoforms
Source: FEBS J. 2009 Mar;276(6):1610–28. doi: 10.1111/j.1742-4658.2009.06893.x (PMC2702505; doi:10.1111/j.1742-4658.2009.06893.x)
Supplement: Supplementary file 8 [file ejb0276-1610-SD8.doc]

**Expression constructs**

The GFP-Human Talin 2 vector (pGFP-TLN2) was constructed as follows. The 5’ end of Human *TLN2* was amplified by PCR from pCR2.1-hTLN2 (gift from Lorenzo Pellegrini, Howard Hughes Medical Institute and Department of Cell Biology, Yale, USA) using the following primers:

5’-ATTCGGATCCATGGTGGCCCTGTCCTTAAA-3’

5’-AATCGTCGACGATATCTGCTCTCCCTCGGT-3’

thus introducing 5’ *Bam*HI and 3’ *Eco*RV-*Sal*I sites (underlined). The fragment was digested with *Bam*HI/*Sal*I and inserted into pEGFP-C1 (Clontech; CMV ubiquitous promoter) digested with *Bgl*l/*Sal*I. Full length GFP-*TLN2* was created by inserting the remainder of the coding region from pCR2.1HT2 as a *Eco*RV/*Kpn*I fragment.

To create pmCherry-TLN2, a two step PCR strategy was employed. First mCherry was amplified from pmCherry (a gift from Bernd Zobiak, University of Hamburg, Germany) using the following primers:

5’-TCAGATCCGCTAGCGCTACCGGTCGCCACCATGGTGAGCAAGGGCGAGGAGGATA-3’

5’-GGGCCACCATGGATCTGAGTCCGGACTTGTACAGCTCGTCCATGCCGCCG-3’

The *TLN2* 5’ region was amplified separately from pGFP-TLN2 using primers:

5’-CGGCGGCATGGACGAGCTGTACAAGTCCGGACTCAGATCCATGGTGGCCC-3’

5’-AATCGTCGACGATATCTGCTCTCCCTCGGT-3’

As well as introducing convenient restriction enzyme sites (underlined), a sequence overlap between the two PCR products was created. Following a second round of PCR in which the two templates were joined together, the product was digested with *Nhe*I/*Eco*RV and cloned into the pGFP-TLN2 vector.

The testis short isoform cDNA was amplified from the first ATG encoded by exon 25c (see text and figure 2) using Clone FANTOM 4931402J01 (genebank AK029828) as a template with the following primers:

5’-ACTTCCGGAGACTTAAAGATAGATGTTTCACAAGAGAAT-3’

5’-GCGAATTCTTAGCCCTCATCTTCCCTCAGCTCTG-3’

This introduced a 5’ *Bsp*EI and a 3’ *Eco*RI restriction site (underlined).

The 5 kb fragment was digested with *Bsp*EI and *Eco*RI and cloned into pEGFP-C1 digested with the same enzymes to give pETS-M1. All the constructs were sequenced to ensure the absence of mutations. pEGFP-C1 was used as a transfection control producing EGFP alone under the control of the CMV promoter.

**Transfection of NIH3T3 cells**

80% confluent NIH3T3 cells were cultured in 90% Dulbecco's modified Eagle medium (DMEM) with 4 mM L-glutamine, 1.5 g/l sodium bicarbonate, 4.5 g/l glucose, 10% Fetal Bovine Serum and penicillin/streptomycin. Transfection was performed using a microporator (MP-100; Digital Bio, Seoul, Korea) according to the manufacturer’s recommendations with 5x10^6^ cells and 10 μg of each construct per 100 μl tip. The electroporation parameters were: 1350 volts, 3 pulses, 20 ms pulse width. Each transfection was performed in duplicate. The cells from one transfection were plated on 12 mm round coverslips in 12-well plates and cultured for 24 hours before fixation for microscopy, and the second transfection was plated on 6 cm plastic dishes for protein analysis after 48 hours in culture (see below).

**Cell fixation, staining and microscopy**

Cells on glass coverslips were fixed in 4% paraformaldehyde for 5 minutes at room temperature, rinsed 3 times in 1x PBS and permeabilised in 1x PBS, 0.1% Triton-X-100 for 5 minutes. Phalloidin-Texas Red (Invitrogen) was applied at a 1:200 dilution for 20 minutes, and followed by washing 3 times in 1x PBS. Nuclei were counterstained with a solution of DAPI (1:5000) in 1x PBS for 2 minutes and the cells were washed 3 more times in 1x PBS before mounting in MOWIOL 4-88 reagent (Calbiochem) containing 1% 1,4-Diazabicyclo [2,2,2] octan (Sigma) as an anti-fading agent. Cells were imaged with an Upright Olympus BX61 automated microscope, 12 Bit camera, X-cite120 fluorescence illumination system, and a multi-slide stage using the relevant filters for the detection of GFP, mCherry and DAPI.

**Protein lysates and Western blotting**

48 hours after transfection, cells were lysed using 100 μl RIPA buffer (50mM Tris, pH8.0, 150 mM NaCI, 0.1% SDS, 1.0% NP-40, 0.5% Sodium Deoxycholate, complete with Protease Inhibitor Cocktail and calpain II inhibitor E64d). Protein concentration in cell lysates was determined using the Bradford assay, and 20 μg of protein extract were run in Laemmli buffer on 6% SDS-PAGE gels and transferred and probed as described in the manuscript. All the antibodies are described in the main Material and Methods.
