# Supplementary material for: Talin 2 is a large and complex gene encoding multiple transcripts and protein isoforms
Source: FEBS J. 2009 Mar;276(6):1610–28. doi: 10.1111/j.1742-4658.2009.06893.x (PMC2702505; doi:10.1111/j.1742-4658.2009.06893.x)
Supplement: Supplementary file 9 [file ejb0276-1610-SD9.doc]

| Primer | Sequence 5’-3’ |
| --- | --- |
| a | GCTCCGCAATGAGACAGTAGCT |
| b | GGATAGGGCCACCATTTTCA |
| c | TCTCGGCTCCTCTCTGCTTGATGTA |
| d | GATCTGCAGCTTGCTCGATA |
| e | CACATCCCGAGCAGAGTCTA |
| f | AATGCCGCAGACACCCGATCGA |
| g | ACCAAGATTGGTTCCTGCGCCT |
| h | CTGGAAAAGTGTGCTCAGGA |
| i | GAAGGCACACCTCCAGAACCAAAG |
| j | GCTCCCTCTTGGTGTAGCTGTCTG |
| k | CTGTGATCGGCAAAGGTCTCGCC |
| l | GGCAGCAACCAGCAGTCTCTGTGAG |
| m | TAATGGTCACTGATGCTGGTGGGAAAA |
| n | TGAGCAATGCCTCCCACGAACTT |
| Ex-6 For | CTCGGTCCTCGGGTTTCCAGTAACA |
| Ex-6 Rev | TGTTACTGGAAACCCGAGGACC |
| Ex-5 For | TGTGCTGCTGTAGGGTAACATCTGGA |
| Ex-5 Rev | CATCCAAGCACCAGAGGAGCCTCTA |
| Ex-4 For | AGTCTCTTGAAGGAGGCAGAAC |
| Ex-4Rev | CCTCCTTCAAGAGACTCCCGGGATA |
| Ex-3 For | GATGATCTAGAAGAGGCGACAGGAGACA |
| Ex-3 Rev | CCTGCTCAAACACACTCTCTTCGGG |
| Ex-2 For | GTATTGCTGCTAGAAGCTTCTCACTCCTCAG |
| Ex-2 Rev | CGGTTTCCCTGAAAAGATCCTGAGG |
| Ex-1 For | GAGCCAACTGGGTGCCAACA |
| Ex-1 Rev | AATGGTGTTGGCACCCAGTTGG |
| Ex 0 For | GCTGACGGAGAAAAACAGCAGGAGGACTA |
| Ex 0 Rev | TCCACCCTTCCCAATCCTCTTCGAG |
| mGapdh For | AGGTCGGTGTGAACGGATTTG |
| mGapdh Rev | TGTAGACCATGTAGTTGAGGTCA |
| A | GCTCTGCAATGAGACAGT |
| B | GGACAGGGCCACCATTTTCC |
| hGAPDH For | GAGTCAACGGATTTGGTCGT |
| hGAPDH Rev | GACAAGCTTCCCGTTCTCAG |
| Ex-7 RACE1 | GCGGGGATGCAGCTACTGTCTCA |
| Ex-7 RACE2 | TCATTGCGGAGCGCCAGCCCACG |
| Ex25b RACE | AGCTGCAGATCACATCCCTATG |
| Ex25c RACE | TCTGCTAGTTCAGCATTACCTCATTGGA |
| Ex26 RACE | CACATCCCGAGCAGAGTCTA |
| Ex34b RACE1 | TCAAAGTCACTCAGAGGGAGCTCGAT |
| Ex34b RACE2 | TGTCTGCGGCATTCACTGGAAGT |
| Ex56 3’RACE1 | CCATAATCCAAAGGGCAGAGTTGTGG |
| Ex56 3’RACE2 | CAGCGCCCCCCTGGAGGAAGAGTCA |
| 5’GT4 | AAATTCTGTGACCTCACAGGCT |
| GT4Vec | CAACTGACCTTGGGCAAGAA |
| 3’GT4 | CCAGGATCTACAGAGAGAGACTTGGCG |
| SGT5’ For | CAAGTGTAGTTGTATGGAGAAGATGTGAGGG |
| SGT5’Rev | GACTCAACAATATCACCAGCTGAAGCCTAT |
| SGT3’ Rev | GTGGTGTGGGGAGACGGCTCATTTG |
| Sp6-2 | AGGTACCGGTCCGGAATTCC |
| hT2-3R | TGGAGGCCACACCAACTGCG |

**Table S1 : sequences of the primers used for RT-PCR**

**Debrand el al**
