# Supplementary material for: Talin 2 is a large and complex gene encoding multiple transcripts and protein isoforms
Source: FEBS J. 2009 Mar;276(6):1610–28. doi: 10.1111/j.1742-4658.2009.06893.x (PMC2702505; doi:10.1111/j.1742-4658.2009.06893.x)
Supplement: Supplementary file 10 [file ejb0276-1610-SD10.doc]

| Exon | Size (bp) | Intron size (bp) | Sequence* |
| --- | --- | --- | --- |
| -7 | 122 | 256 | ctccggttccgcgcccggagccagcagcggcggcagcggcggcggcggcggcgcagcgggagcggagctggctgcactcgtgggctggcgctccgcaatgagacagtagctgcatccccgcg |
| -6 | 721 | 73526 | gtttcgccgcactcaagccggttgccgggtttgcaaacagtcttgcagcgggagccagagagctgatccctagccttaagcagctggtcccctcccgggctcggtcctcgggtttccagtaa  cagggtcggtaaagcggcccggtttgcaccgaggggccccttggactccggccgtgaccccggcgccgactgagttggcggagagcagagcggcttcattccagcactggctgccccgactt  aaagcgccactttgcaaacaagtctcccgctctggctcgtggagaaagacaccccgggatgcttcctagtccccgactctgagacctgcagattgcaggactccccctttccctggccgtct  cgctaacaaccagctgctggccgcggaccgttcagggaaagcgcggggcggagagcgagggcagggtttggacccagtgcggcggcctctcgtctccttccccgaccctcaggagctgagat  ttcgcgccggcggggatcgggagggatctggagactggcttttcggggcccaaggaacgacgttcgagggcgccatattttcagtgggtttttacagaggctaaggggggcgcccactttcc  gaagcccccgggggaagccggagcgattgtcgttgggaaagtttgcgcgcccgggcgagctaagaggcggctgtccccacaagggcgcgcggactgcagcgcgtcgcccag |
| -5 | 90 | 19857 | tgtgctgctgtagggtaacatctggactggaatagaggctcctctggtgcttggatgtccctaagttcctggatcggcttgttgaagact |
| -4 | 35 | 174 | tatcccgggagtctcttgaaggaggcagaacccag |
| -3 | 72 | 79685 | gatgatctagaagaggcgacaggagacaccccctcccccttcccgaagagagtgtgtttgagcaggtttcag |
| -2 | 74 | 30989 | gtattgctgctagaagcttctcactcctcaggatcttttcagggaaaccgaaactgagtaaactgagaatttag |
| -1 | 42 | 24545 | aagttagctgagccaactgggtgccaacaccattggatgtgg |
| 0 | 99 | 6568 | cctttgctgacggagaaaaacagcaggaggactaaaaggtagactcgaagaggattgggaagggtggatgacttattcagctatccttgaacatcaatg |
| 1 | 172 | 1960 | atgttctgagtgaaacccatcaagtcatctatgaaa**ATG**GTGGCCCTATCCTTAAAGATCTGTGTCCGCCACTGCAATGTGGTGAAGACCATGCAGTTTGAGCCATCTACAGCTGTGTATGACG  CTTGTCGAGTCATTAGGGAGAGAGTACCTGAGGCACAGACTGGGCAAG |
| 2 | 98 | 1749 | CTTCTGACTATGGGCTGTTTCTCTCCGATGAAGACCCAAGGAAAGGGATTTGGCTAGAAGCAGGTCGGACACTGGATTACTACATGTTGCGGAATGGG |
| 3 | 130 | 970 | GACATTCTGGAGTACAAGAAGAAACAGAGGCCTCAGAAAATCCGAATGTTGGATGGCTCTGTGAAGACGGTGATGGTAGATGACTCCAAGACGGTGGGGGAGCTCCTGGTCACGATCTGCAGCA  GGATAG |
| 4 | 153 | 3739 | GAATAACAAATTATGAAGAATACTCTTTAATCCAAGAAACCATTGAAGAAAAGAAAGAAGAAGGGACAGGCACACTAAAAAAAGATAGGACGTTGTTACGAGATGAGAGGAAGATGGAGAAGCT  GAAGGCCAAACTGCACACAGATGATGACT |
| 5 | 143 | 1885 | TAAATTGGCTAGATCACAGCCGGACATTCAGAGAGCAAGGCGTGGATGAAAATGAAACATTGTTGCTCCGACGGAAGTTCTTTTACTCTGATCAGAATGTGGACTCGAGAGACCCGGTGCAGTT  GAACTTGCTTTATGTCCAG |
| 6 | 128 | 9686 | GCTCGGGATGACATCCTGAATGGCTCTCACCCTGTCTCCTTCGAGAAGGCCTGTGAGTTTGGTGGGTTCCAAGCCCAGATACAGTTTGGACCTCACGTGGAACACAAACACAAGCCTGGATTCT  TAGA |
| 7 | 64 | 974 | TCTAAAGGAGTTCCTGCCAAAAGAGTACATCAAGCAGAGAGGAGCCGAGAAGCGAATATTTCAG |
| 8 | 100 | 4960 | GAACATAAAAACTGTGGAGAGATGAGTGAAATAGAAGCCAAGGTGAAGTACGTCAAACTCGCCCGGTCCCTCCGGACATATGGGGTGTCCTTTTTCCTAG |
| 9 | 156 | 3380 | GAAAAAATGAAAGGCAAGAACAAGTTGGTTCCCCGTCTCCTGGGCATCACCAAGGACTCCGTCATGCGTGTGGATGAGAAGACCAAGGAGGTGCTTCAGGAGTGGCCTCTCACCACGGTCAAGCG  CTGGGCCGCTTCACCCAAGAGCTTCACACTG |
| 10 | 102 | 1148 | GATTTTGGGGAGTACCAAGAGAGCTACTATTCAGTACAAACCACAGAGGGGGAGCAAATATCCCAGCTGATTGCAGGATACATTGATATCATCCTCAAAAAG |
| 11 | 77 | 3087 | AAACAAAGCAAAGATAGATTTGGCCTGGAAGGGGATGAGGAGTCAACTATGTTGGAAGAATCAGTTTCCCCAAAAAA |
| 12 | 181 | 1201 | ATCTACCATCCTGCAGCAGCAGTTCAACCGGACTGGGAAAGCAGAGCATGGTTCGGTAGCACTGCCAGCTGTCATGCGCTCTGGCTCCAGTGGGCCTGAGACGTTCAATGTTGGCAGCATGCCGTC  ACCACAGCAGCAGGTCATGGTCGGACAGATGCACCGAGGACACATGCCTCCGCTG |
| 13 | 114 | 4499 | ACGTCGGCACAGCAAGCCCTTATGGGGACCATCAACACAAGCATGCACGCTGTCCAGCAGGCTCAGGATGACCTCAGCGAGCTCGACTCGCTGCCACCCCTCGGCCAAGATATG |
| 14 | 109 | 1388 | GCATCTAGGGTATGGGTTCAGAACAAAGTGGATGAATCCAAGCATGAAATCCACTCTCAAGTCGATGCTATCACAGCTGGGACAGCTTCAGTTGTCAACCTCACAGCTG |
| 15 | 209 | 518 | GTGACCCTGCAGACACTGACTACACAGCCGTGGGCTGTGCAATCACTACCATTTCTTCCAACCTGACCGAAATGTCCAAGGGGGTCAAGCTGCTGGCAGCCCTCATGGACGATGACGTGGGCAGTGG  GGAGGATCTTCTCAGAGCTGCCAGGACCCTCGCCGGGGCTGTGTCTGATTTGCTGAAGGCTGTGCAGCCTACTTCAGGAGAG |
| 16 | 99 | 6115 | CCTCGACAGACTGTTTTGACTGCTGCCGGAAGCATCGGCCAAGCCAGTGGAGATCTCCTCAGACAGATTGGAGAGAACGAGACGGATGAGAGATTCCAA |
| 17 | 168 | 1506 | GATGTTTTAATGAGTCTAGCTAAGGCTGTTGCCAATGCTGCTGCCATGTTAGTACTGAAGGCGAAGAATGTGGCCCAGGTGGCTGAAGACACCGTCCTACAGAACAGGGTGATCGCCGCAGCCACCCA  GTGTGCCCTCTCTACCTCGCAGCTTGTGGCGTGTGCTAAG |
| 18 | 295 | 2088 | GTGGTGAGCCCCACCATCAGCTCTCCCGTGTGCCAGGAGCAGCTGATCGAAGCTGGGAAGCTGGTGGACCGCTCTGTGGAAAACTGTGTCCGAGCCTGCCAGGCGGCCACCAGTGATAGCGAGCTCCT  GAAGCAGGTCAGCGCGGCGGCCAGTGTGGTCAGCCAGGCCCTGCACGACCTCCTGCAGCATGTGCGGCAGTTCGCCAGCCGAGGCGAGCCCATTGGCCGCTATGACCAGGCCACCGACACCATCATGT  GTGTCACTGAGAGCATCTTCAGTTCCATGGGCGATGCTG |
| 19 | 167 | 1902 | GTGAGATGGTGCGCCAAGCCCGGGTGCTAGCCCAGGCCACATCAGATCTCGTCAATGCCATGAGGTCAGATGCGGAGGCTGAGATTGACATGGAGAATTCCAAGAAGCTCCTGGCGGCCGCAAAGCTC  TTGGCTGACTCCACTGCCCGCATGGTGGAAGCTGCGAAG |
| 20 | 129 | 1639 | GGGGCTGCAGCCAACCCAGAGAATGAAGACCAGCAGCAGAGGCTGAGAGAAGCTGCCGAGGGTCTCCGAGTAGCAACCAATGCCGCTGCGCAGAATGCTATTAAGAAAAAAATCGTCAACCGGCTGGAG |
| 21 | 114 | 1887 | GTTGCAGCCAAGCAGGCTGCAGCTGCTGCCACACAGACCATCGCAGCCTCCCAGAATGCAGCCATCTCCAACAAGAACCCCTCAGCCCAGCAACAACTGGTCCAGAGCTGCAAG |
| 22 | 114 | 3885 | GCGGTGGCTGATCACATCCCTCAGCTAGTGCAGGGCGTGAGAGGGAGCCAAGCACAAGCAGAAGACCTCAGTGCCCAGCTGGCTCTCATCATCTCCAGCCAGAACTTCCTGCAG |
| 23 | 135 | 2329 | CCTGGAAGCAAGATGGTGTCCTCTGCTAAGGCGGCGGTGCCCACTGTGAGTGACCAGGCTGCAGCCATGCAGTTGAGTCAGTGTGCCAAGAACCTGGCAACCAGCCTGGCCGAGCTGCGCACAGCCTCGC  AGAAG |
| 24 | 129 | 1081 | GCCCACGAAGCCTGTGGCCCCATGGAAATAGATTCTGCACTGAACACTGTGCAGACTCTCAAGAATGAGCTGCAGGACGCCAAGATGGCCGCTGCAGAAAGTCAACTGAAACCACTTCCAGGAGAAACG |
| 25 | 114 | 7366 (to 26) | CTGGAAAAGTGTGCTCAGGACCTGGGAAGCACCTCTAAGGGAGTGGGCTCCTCCATGGCACAGCTCCTGACCTGTGCTGCTCAAGGCAATGAGCATTACACAG |
| 25b | 77 | 2631 | ggtggatagtcacatagggatgtgatctgcagcttgctcgatagctctcgggagagggggcttggatggaagtgcag |
| 25c | 172 | 1603 | tgtgctaaggcctgagggcctcagattgtgtttattccaatgaggtaatgctgaactagcagaggactgctcatctgcatcaaa**ATG**GACTTAAAGATAGATGTTTCACAAGAGAATATTCAACGGCCAT  TAAATGTATTTATTAAGTGCTCTCAACATCATTACCATACGA |
| 26 | 209 | 1014 | GCGTGGCAGCAAGAGAAACAGCCCAGGCCCTGAAAACACTGGCCCAGGCTGCCCGGGGAGTGGCGGCATCCACAA**ATG**ATCCTGAGGCTGCTCATGCCATGTTAGACTCTGCTCGGGATGTGATGGAGGG  CTCTGCCATGCTCATCCAGGAAGCCAAGCAGGCACTAATTGCACCTGGAGACACAGAGAGTCAGCAGAGATTAGCCCAG |
| 27 | 120 | 2324 | GTGGCCAAAGCTGTGTCTCACTCACTGAATAACTGCGTGAATTGCCTTCCTGGACAGAAGGATGTGGATGTCGCCTTGAAGAGCATCGGGGAGGCCAGCAAGAAGCTACTTGTAGATTCG |
| 28 | 198 | 994 | CTACCTCCAAGCACAAAGCCCTTCCAGGAGGCCCAGAGTGAGCTGAACCAGGCTGCTGCTGATCTGAACCAATCTGCTGGGGAAGTCGTCCATGCCACAAGGGGCCAGAGTGGAGAGCTGGCAGCCGCTTC  TGGAAAATTCAGTGATGACTTTGACGAGTTCCTGGATGCCGGCATTGAGATGGCTGGCCAGGCACAG |
| 29 | 140 | 6288 | ACAAAAGAAGACCAGATGCAAGTGATAGGGAACCTGAAGAATATCTCGATGGCGTCCAGCAAGCTCCTGCTGGCCGCCAAGTCTCTCTCTGTAGATCCTGGAGCTCCCAACGCAAAAAATCTCCTGGCTGC  AGCTGCAAG |
| 30 | 94 | 2768 | AGCTGTGACAGAGAGCATCAATCAGCTCATCATGCTGTGTACTCAGCAGGCCCCTGGGCAGAAGGAGTGTGACAACGCCCTGCGTGAGCTCGAG |
| 31 | 90 | 2327 | ACTGTCAAGGGGATGTTGGAAAACCCGAATGAGCCTGTGAGCGACCTCTCTTATTTTGACTGCATTGAGAGTGTGATGGAGAACTCCAAG |
| 32 | 123 | 1440 | GTTCTGGGCGAGTCAATGGCAGGGATTTCACAGAATGCCAAGACTGGAGACCTCCCTGCCTTCGGAGAATGTGTGGGGATTGCATCTAAGGCTCTCTGTGGGCTGACAGAGGCAGCAGCCCAG |
| 33 | 144 | 2200 | GCTGCGTATCTGGTTGGCATCTCTGATCCGAACAGCCAGGCAGGTCACCAAGGCCTGGTGGACCCCATCCAGTTTGCCAGGGCTAACCAGGCGATCCAGATGGCATGTCAGAACTTGGTGGACCCTGGCAGC  AGCCCATCACAG |
| 34 | 162 | 3135 (to 35) | GTTCTGTCGGCTGCCACTATTGTTGCCAAGCACACATCAGCCTTATGCAATGCCTGCCGCATCGCCTCATCCAAGACGGCCAACCCTGTTGCCAAGCGGCACTTTGTCCAGTCAGCCAAGGAGGTTGCCAAC  AGCACCGCCAACCTGGTCAAGACCATCAAG |
| 34b | 89 | 2757 | AGCCCCCACCCAGAAGTCAGTCAGAGTGAGCCTTGCAGAGGCCAAAGGAAAGAGCATCCATCCGGCCTGTGGCAGCTGGAAGCTGGCTG |
| 35 | 141 | 485 | GCCCTGG**ATG**GGGATTTCTCTGAAGACAATCGCAATAAGTGTCGAATTGCTACCACGCCCTTGATCGAGGCGGTGGAGAACCTAACAGCGTTTGCATCAAACCCCGAGTTTGCCAGCATTCCTGCACAGATCA  GCTCTGAG |
| 36 | 141 | 1697 | GGCTCCCAGGCGCAGGAACCAATCTTGGTCTCAGCCAAGACCATGCTGGAGAGCTCATCGTACCTCATCCGCACCGCGCGCTCTCTGGCCATCAACCCCAAAGATCCGCCCACCTGGTCCGTGTTGGCTGGAC  ACTCCCACACTGTGTCTGACTCCATTAAGAGTCTCATCACGTCTATCAG |
| 37 | 133 | 3721 | GGACAAAGCCCCAGGGCAGAGGGAATGTGACTACTCCATTGATGGCATCAACCGGTGCATCAGGGACATCGAGCAG GCCTCCCTGGCCGCGGTCAGCCAGAGCCTGGCCACGAGGGATGACATCTCTGTGGAG |
| 38 | 100 | 3353 | GCCCTACAGGAGCAGCTGACCTCAGTGGTCCAAGAAATCGGACATCTTATTGATCCCATTGCCACAGCTGCCCGAGGAGAAGCTGCCCAACTTGGACATA |
| 39 | 171 | 7259 | GTGACACAGCTGGCGAGCTACTTTGAGCCCTTGATCTTAGCTGCAGTTGGTGTCGCCTCCAAGATGCTGGACCATCAACAACAGATGACAGTGCTGGACCAGACCAAGACTCTTGCAGAGTCTGCCCTGCAGAT  GCTGTATGCAGCCAAGGAAGGTGGAG GTAACCCCAAG |
| 40 | 147 | 2560 | GCACAGCACACCCACGACGCCATCACAGAGGCTGCGCAGCTGATGAAGGAAGCTGTGGATGACATCATGGTGACACTGAATGAAGCAGCCAGTGAGGTGGGACTGGTGGGAGGCATGGTGGACGCCATCGCAGA  GGCTATGAGCAAG |
| 41 | 100 | 2591 | CTGGATGAAGGCACACCTCCAGAACCAAAGGGAACGTTTGTTGACTACCAGACGACTGTGGTTAAATACTCCAAAGCCATTGCCGTCACAGCTCAGGAAA |
| 42 | 120 | 5686 | ATGACTAAGTCGGTTACTAACCCGGAGGAGTTGGGAGGCCTGGCTTCACAAATGACCACTGACTATGGGCACCTGGCTCTCCAGGGCCAGATGGCAGCAGCCACCGCCGAACCAGAGGAG |
| 43 | 147 | 842 | ATTGGATTCCAGATTCGCACACGTGTGCAGGACCTTGGCCATGGCTGTATCTTCCTGGTGCAAAAGGCAGGAGCCCTCCAGGTGTGCCCCACAGACAGCTACACCAAGAGGGAGCTGATCGAGTGTGCCCGCTCT  GTCACTGAGAAG |
| 44 | 167 | 2777 | GTGTCCTTGGTGCTGTCTGCTCTCCAGGCTGGAAACAAGGGGACCCAGGCATGCATCACGGCCGCAACTGCAGTTTCTGGGATCATTGCCGATCTGGACACCACCATTATGTTTGCAACAGCTGGCACACTGAAT  GCAGAGAACGGCGAGACCTTTGCCGATCACAG |
| 45 | 184 | 739 | GGAGAACATTCTGAAGACAGCCAAGGCCTTGGTGGAAGACACAAAGCTCCTCGTGTCTGGGGCTGCATCCACTCCAGACAAGCTGGCCCAGGCCGCTCAGTCCTCAGCAGCCACCATCACCCAGCTGGCAGAGGT  GGTCAAGCTGGGGGCCGCCAGCCTGGGCTCCAATGACCCTGAGACCCAG |
| 46 | 126 | 3303 | GTGGTACTGATAAATGCCATCAAAGACGTGGCCAAGGCCCTTTCCGATCTCATTGGTGCTACCAAGGGAGCTGCCAGCAAGCCGGCTGACGACCCCTCCATGTACCAGCTCAAAGGAGCCGCCAAG |
| 47 | 117 | 157 | GTGATGGTGACCAATGTCACCTCCCTTCTTAAGACTGTGAAGGCAGTGGAAGACGAGGCCACCCGGGGCACAAGGGCACTTGAGGCCACCATCGAGTACATAAAACAGGAGCTCACA |
| 48 | 186 | 2143 | GTGTTCCAGTCAAAAGACATACCTGAAAAGACATCATCACCCGAGGAATCAATAAGAATGACGAAAGGTATCACCATGGCAACAGCGAAAGCCGTGGCAGCTGGGAACTCATGTAGACAAGAGGATGTAATTGCA  ACCGCCAATCTGAGCAGGAAGGCTGTCTCAGATATGTTGATAGCTTGCAAG |
| 49 | 108 | 5208 | CAAGCATCCTTCTACCCTGATGTCAGTGAAGAGGTGCGAACCAGAGCCTTGCGGTATGGCACAGAATGCACCTTGGGCTACCTGGACCTACTAGAACACGTCTTGGTG |
| 50 | 106 | 8089 | ATCCTTCAAAAGCCAACCCCGGAACTCAAGCATCAGCTGGCTGCTTTCTCCAAGAGAGTTGCTGGAGCTGTGACGGAGCTCATCCAGGCAGCGGAAGCCATGAAAG |
| 51 | 125 | 1522 | GAACAGAGTGGGTGGATCCAGAAGACCCAACCGTCATTGCAGAAACTGAGTTACTGGGAGCTGCAGCATCCATTGAAGCCGCTGCCAAGAAGTTAGAGCAGCTGAAGCCAAGAGCAAAGCCAAAG |
| 52 | 126 | 13187 | CAAGCAGACGAGACCCTGGATTTTGAAGAACAGATCTTAGAAGCTGCTAAATCCATTGCTGCTGCCACAAGTGCCCTGGTCAAATCGGCCTCAGCAGCCCAGAGGGAGCTGGTAGCCCAAGGCAAG |
| 53 | 63 | 91 | GTGGGCTCCATCCCTGCCAATGCTGCCGATGATGGACAGTGGTCCCAGGGGCTGATCTCTGCC |
| 54ab | 183+45 | 2961 | GCCCGGATGGTGGCGGCAGCAACCAGCAGTCTCTGTGAGGCAGCCAATGCCTCTGTGCAGGGACATGCCAGTGAGGAAAAGCTCATCTCATCCGCCAAGCAGGTCGCAGCTTCAACCGCTCAGCTGCTGGTGGCCT  GCAAGGTGAAGGCCGACCAGGATTCAGAGGCCATGAAGCGGCTACAGGTAATGGTCACTGATGCTGGTGGGAAAATACTCCTGTTGGAGCGG |
| 55 | 126 | 2457 | GCGGCAGGAAATGCTGTGAAAAGAGCCTCAGACAATCTTGTTCGTGCAGCCCAGAAGGCAGCATTTGGCAAAGCTGATGATGACGATGTTGTGGTGAAAACCAAGTTCGTGGGAGGCATTGCTCAG |
| 56 | 847 | NA | ATCATTGCAGCCCAGGAGGAAATGCTGAAGAAAGAACGAGAGCTGGAGGAAGCCAGGAAGAAGCTGGCCCAGATCCGCCAGCAGCAGTACAAATTCCTCCCCACAGAGCTGAGGGAAGATGAGGGCTAAgacaccc  cccatgctggtgagccccaggaggtggttcctactcccccaacaagaactggacatactggtgtcccccagagccaggaacttcctggaaactgctggccctccaccggagtctgtctctctcagtcctcagtccc  gtcactatcagctgcatgatcatggtctcacacggtacagtgtcccacccacagctcttccagctcccctcacgcctcgctatatctcaggacagagggcgcatttcatggactgttaccaaaaaagaaaagtcag  tattacgttgttctcagacacctgtactccgtgtcctctaagactgctccagggcctctttgtgaaatcacactcgggaaagaacaatgctaataacggctcacaggaatcatcagtatcatggacatagagattc  tctctctggagagaagacagctggagttggacactgttaataaagccagaaacactcccaagtgaactctggagtcacagaggtctgtgtaagtgtggggcactgactcacccacgggggagaagatcccctaccc  gtttgcacgcttttctcgcctcagtgtgtaagctccttgtacaactacacccatcttgtattctgtggcccagaaaaatggaagttatttttttttcccccttccataatccaaagggcagagttgtggaaagtgg  tcagggttaggtgggtaggggccggaataaa |

* lower case: non coding

upper case: coding

**ATG**: first codon

aataa: polyadenylation signal

**Table S2**: **list of mouse Tln2 exons**

**Debrand et al**
